# *Ex Vivo* Irradiation of Lung Cancer Stem Cells Identifies the Lowest Therapeutic Dose Needed for Tumor Growth Arrest and Mass Reduction *In Vivo*

**DOI:** 10.3389/fonc.2022.837400

**Published:** 2022-05-12

**Authors:** Caterina Puglisi, Raffaella Giuffrida, Giuseppina Borzì, Salvatore Illari, Francesco Paolo Caronia, Paolo Di Mattia, Cristina Colarossi, Gianluca Ferini, Emanuele Martorana, Giovanni Sette, Adriana Eramo, Aurelio Lorico, Alfio Di Grazia, Stefano Forte

**Affiliations:** ^1^ Genomics and Experimental oncology unit, IOM Ricerca, Viagrande, Italy; ^2^ Operative Unit of Radiotherapy, Rem Radioterapia, Viagrande, Italy; ^3^ Unit of Radiotherapy, Fondazione IOM, Viagrande, Italy; ^4^ Unit of Thoracic Surgery, Azienda di Rilievo Nazionale ad Alta Specializzazione (ARNAS) Civico, Palermo, Italy; ^5^ Department of Experimental Oncology, Mediterranean Institute of Oncology, Viagrande, Italy; ^6^ Department of Oncology and Molecular Medicine, Istituto Superiore di Sanità, Rome, Italy; ^7^ Department of Basic Sciences, Touro University Nevada College of Medicine, Henderson, NV, United States

**Keywords:** lung cancer, cancer stem cells, personalized radiotherapy, radiotherapy-induced toxicity, *in vitro* modeling

## Abstract

Radiotherapy represents a first-line treatment for many inoperable lung tumors. New technologies offer novel opportunities for the treatment of lung cancer with the administration of higher doses of radiation in smaller volumes. Because both therapeutic and toxic treatment effects are dose-dependent, it is important to identify a minimal dose protocol for each individual patient that maintains efficacy while decreasing toxicity. Cancer stem cells sustain tumor growth, promote metastatic dissemination, and may give rise to secondary resistance. The identification of effective protocols targeting these cells may improve disease-free survival of treated patients. In this work, we evaluated the existence of individual profiles of sensitivity to radiotherapy in patient-derived cancer stem cells (CSCs) using both *in vitro* and *in vivo* models. Both CSCs *in vitro* and mice implanted with CSCs were treated with radiotherapy at different dose intensities and rates. CSC response to different radiation doses greatly varied among patients. *In vitro* radiation sensitivity of CSCs corresponded to the therapeutic outcome in the corresponding mouse tumor model. On the other side, the dose administration rate did not affect the response. These findings suggest that *in vitro* evaluation of CSCs may potentially predict patients’ response, thus guiding clinical decision.

## Introduction

Lung cancer is the leading cause of cancer-related death worldwide (1–3) and the second most common cancer diagnosis by sex, following prostate cancer for men and breast cancer for women (3). The known risk factors for lung cancer include behavioral, environmental, and genetic risk factors, all having a role in tumor development and also affecting the individual capacity for response. Lung cancer has one of the lowest survival rates, along with liver and pancreatic cancer. The overall 5-year survival rate for lung cancer has not changed significantly in the last decades (3–5), going from 12% for lung cancers (all stage combined) diagnosed from 1975 to 1977 to 18% from 2003 to 2009 (5, 6). Advanced lung cancer has extremely poor prognosis, with a 5-year survival of only 5% (3).

Non–small cell lung cancer (NSCLC) accounts for over three quarters of all lung cancer cases. Thoracic surgery is considered the standard of care for people with early-stage lung cancer. However, less than 20% of patients with NSCLC are suitable for surgery (7). Radical radiotherapy is the first option for patients with unresectable tumors, with the aim to provide long-term local disease control. The long-term survival rate with radiotherapy is around 15% at 5 years (8). The radiotherapy protocol usually consists in the administration of at least 60 Gy in 30 daily fractions of 2 Gy delivered in 6 weeks (9). An alternative schedule for radical radiotherapy delivering 55 Gy in 20 fractions has also been employed (10).

Stereotactic ablative radiotherapy (SABR) is meant to deliver large doses of radiation with a high precision of 1–2 mm to small lesions (less than 1 cm3) (11, 12), and it has been shown that it provides a survival benefit superior to that of conventional radiotherapy with curative intent (13). It has also been suggested that, under specific circumstances, SABR is safer than surgery while providing a comparable efficacy profile (14, 15). On the other side, different toxicities, ranging from mild to severe, have been reported in lung cancer patients treated with SABR (16–22). It has been demonstrated that the frequency of severe effects is influenced by both the tumor volume and the administered dose (17). Moreover, the availability of new technology like the flattening filter‐free (FFF) beams provides the opportunity to optimize treatment delivery in SABR through the administration of high doses in a shorter time frame. Although the practical benefit of higher dose rates is evident (reduced treatment and immobilization time by more than 50%, less physician‐ordered image guidance, etc.) (23), contrasting evidence has been reported on the efficacy side (24–27).

Many recent studies show that targeting cancer stem cells (CSCs) is a promising therapeutic strategy, because of the ability of these cells to initiate and sustain tumor growth and to generate the heterogeneous cell population forming the entire tumor. The existence of CSCs has been described in both hematological and solid tumors (28–38), including lung cancer (39). The biology of CSCs is closely associated with tumorigenesis and therapeutic resistance. Indeed, conventional anti-cancer therapies, which are able to kill the majority of differentiated tumor cells, may spare CSCs, which remain unaffected and may be responsible for tumor recurrence and progression. Hence, CSCs represent the primary therapeutic target for complete tumor eradication.

We recently proposed a model for the prediction of response to radiotherapy in rectal cancer using *in vitro* irradiation of patient-derived CSCs (40).

In this work, we assessed the specific response of CSCs to different radiotherapy doses and dose rate combinations. To this aim, CSCs isolated from tumor tissues of lung cancer patients were exposed to gamma-radiation using different protocols to test their *ex vivo* radiosensitivity. Subsequently, animal models derived from CSCs xenotransplantation, resulting in tumors with a well-differentiated morphology reminiscent of human lung cancers, were generated and subjected to the same protocols of radiotherapy to verify whether *ex vivo* sensitivity was also reproduced *in vivo*.

Our data show a significant correspondence between *ex vivo* and *in vivo* CSCs sensitivity to the different radiotherapy protocols tested, suggesting that the cellular model may be sufficient to assess the suitability of treatment. Our findings provide a feasible translational approach for the prediction of radiotherapy curative efficacy.

## Materials and Methods

### Primary Human Tumor Material

Tumor samples were obtained in accordance with consent procedures approved by the institutional ethics committee of P.O. **“**Civico**”** Hospital of Palermo (Italy) (authorization document no. 26, protocol 239 of October 9, 2018). Tumor tissue dissociation and procedures for medium preparation and expansion of lung CSCs *in vitro* were performed as previously described (39, 41).

### Tumor Cell Isolation and Culture

Primary spheroid cultures used in this study were derived from lung cancer patients. Patient’s clinical characteristics are reported in **Table 1**.

**Table 1:** Patient’s clinical characteristics.

LCSC	Patient sex/age	Tumor subtype	TNMstage/grading	Diagnostic markers expression
Line 36	M/70	SSC	pT2pN2pMX (IIIA)-G2	p63+, CK7−, TTF1−
Line 136	M/58	SSC	pT3pN0pMx-IIB-G3	p63+, CK7−, TTF1−
Line 196	F/75	SSC	pT2N0-IB	p63+, CK7−, TTF1−
Line 229	F/65	AC	pT4pN1IIIA-G3	TTF1+, ALK−, ROS1−

LCSC, lung cancer stem cell; SSC, squamous cell carcinoma; AC, adenocarcinoma.

Tissue samples were collected by a surgeon or a pathologist immediately after each patient’s surgery, quickly washed two to three times in cold saline buffer, then transferred in Dulbecco’s modified Eagle’s medium (Thermo Fisher Scientific, Carlsbad, CA, USA) containing 5% penicillin-streptomycin-amphotericin B solution (Lonza Group) and kept in this medium at 4°C until processing within 24–48 h. CSC isolation from biopsies was performed as previously described (37, 38). Tissues were first extensively washed in PBS (Gibco, Thermo Fisher Scientific, Carlsbad, CA, USA) and then subjected to mechanical and enzymatic digestion with Collagenase type II (Thermo Fisher Scientific) and DNAse I (Roche Diagnostics, Indianapolis, IN, USA) at 37°C for 1 h. Cell suspension was then filtered through a 100-μm nylon; the cell pellet was resuspended in CSC medium (Tumorsphere Medium XF, PromoCell, Heidelberg, Germany), plated in ultra-low attachment tissue culture flasks (Corning Costar, Cambridge, MA, USA) and incubated at 37°C in a humidified atmosphere of 5% CO_2_. Every 2–3 days, half of the culture medium was replaced with fresh medium. Under this stringent culture condition, immature cells grow slowly and form non-adherent clusters, called tumor spheres, whereas non-malignant cells or differentiated cells die. Tumor spheres became evident after a variable length of time, ranging from 5 to 7 days to 3 weeks. Regular culture splitting (1:2) was usually needed after 3–6 weeks from isolation. Spheroids were weekly subjected to mechanical or enzymatic dissociation by incubation for 10 min at room temperature with Accutase enzyme (Gibco).

Primary spheroids were biobanked at early passages after isolation, characterized for common genetic mutations, and validated for their ability to reproduce the histology of the original tumor in mouse xenografts, as previously demonstrated and reported in **Table 2** and **Figure 4** (39, 41–44). Their phenotype, molecular pattern, and ability to reproduce a patient-like tumor in mice are maintained also after repeated passages *in vitro*. The lung cancer stem cell (LCSC) population is heterogenous; however, the proportion of cells with specific phenotypes tends to be maintained in culture (39, 41–44). In addition, because of the possibility to bank several stocks of cells at first passages, we always use LCSC at early passages (lower than 12th passage in this study), guaranteeing the reproducibility of results.

**Table 2:** Histological, molecular, and functional properties of lung cancer CSCs lines.

	Line 229	Line 136	Line 36	Line 196
**DIAGNOSIS**	AC	SCC	SCC	SCC
**CD44**	96.2%	99%	0.8%	52%
**CD133**	4%	2.2%	90%	0%
**SOX2**	LOW	NEGATIVE	HI	LOW
**ALDH**	NEGATIVE	MEDIUM	HI	NEGATIVE
**EGFR**	WT	WT	WT	WT
**P53 (exon 5, 6 an 7)**	WT	WT	WT	WT
**KRAS**	MUTANT	WT	WT	MUTANT

### Evaluation of Surface Markers Expression

The expression of stem cell markers was evaluated by flow cytometry analysis with a FACSAria II [Becton Dickinson (BD), Franklin Lakes, NJ, USA]. Single cells dissociated from spheroids were incubated with the appropriate dilution of specific antibody: anti CD133-PE (phycoerythrin) (Miltenyi Biotec, Bergisch Gladbach, Germany); anti CD44–fluorescein isothiocyanate (FITC) (BD Biosciences), and anti Ep-CAM/BerEP2 (Dako). Unstained cells were used as negative control.

Stemness was also evaluated by using the Aldefluor KIT (STEMCELL Technologies, Vancouver, BC, Canada) according to the manufacturer’s instructions. The ALDEFLUOR™ reagent system is a non-immunological method to identify stem/progenitor cells on the basis of their aldehyde dehydrogenase (ALDH) activity. Expression analyses of stem cell-associated protein SOX2 were performed by immunoblot as previously shown (41).

### *In Vitro* Cell Irradiation

CSCs were seeded into dishes of 35 × 10 mm (Corning, NY, USA) and reached approximately 80% confluency at the time of irradiation.

A system for the *in vitro* irradiation of CSCs and a custom-designed irradiation geometry have been previously described (40). This system allows cell cultures to be treated through the same equipment used for patients (Varian Novalis-TrueBeamSTx linear accelerator). To simulate the flow of radiation beams through human tissues before reaching the cells, each dish containing cultured CSCs was inserted inside a niche of a custom-built phantom made of plexiglass to position the cells at the radiation isocenter. From a physical point of view, this material resembles water, which is the main component of human tissues, when interacting with radiation during treatment. CSCs were irradiated through a Varian Novalis-TrueBeamSTx linear accelerator, a radiotherapy equipment able to perform stereotactic treatments with very high precision, using the high-dose rate FFF technique and the high-definition multilamellar collimator (MLC), with minimum leaves size at the isocenter of 2.5 mm, specifically designed to treat small lesions. To identify the lowest effective dose (LED), each line was treated different doses of radiation: 5, 8, 10, and 20 Gy. For each used dose, three different dose rate configurations [with different frequencies expressed as monitor unit (MU)/min as defined by Holmes et al. (45)] have been used: 600, 1,400, and 2,400 MU/min. The plan consisted of two opposed photon beams of 8 × 8 cm^2^ defined at the machine isocenter located at the center of the niche containing the plate.

After irradiation, cells were incubated at 37°C in a humidified atmosphere of 5% CO_2_ for further 24 h, 48 h, 72 h, 7 days, and 14 days, respectively, and then analyzed for cell viability, apoptosis, and spheres formation capabilities.

### Cell Proliferation Assay

The cell viability assay was performed using the CellTiter96^®^ Aqueous One Solution Cell Proliferation Assay Kit (Promega) according to the manufacturer’s instructions. Fluorescence signal was detected with Synergy HT (Biotech) at 24 h, 48 h, 72 h, 7 days, and 14 days after the treatment.

### Apoptosis Assay

Annexin V staining of phosphatidylserine (PS) in the outer surface of cellular membrane is a widely used assay for studying cellular apoptosis, as an increase of PS staining is directly connected with early apoptosis. Cells (1 × 10^5^) for each sample were stained at 24 h, 48 h, 72 h, 7 days, and 14 days, with Annexin V FITC at a final concentration of 0.375 μg/ml (BD Biosciences), according to the manufacturer’s instructions. To distinguish between early apoptotic cells with intact cellular membranes and necrotic or late-apoptotic cells, 1 μg of propidium iodide (PI) was added to each sample. We performed cytometric analysis with a FACSAria II flow cytometer (BD Biosciences). For each measurement, 1 × 10^4^ cells were counted and results analyzed. Three replicates were analyzed for each CSC line in each condition assessed.

### Sphere Formation Assay

CSCs were dissociated with accutase (Gibco) and resuspended in fresh medium to generate a single-cell suspension with a density of 100 cells/200 μl. Then, 200 μl of single-cell suspension was dispensed into each well in a 96-well non-treated plate. Cell cultures were maintained in medium and checked after 14 days to establish their clonogenic potential. The number of floating culture aggregates (spheres) was compared, for each treated line, with the corresponding untreated culture and reported as percentage.

### Animal Models

All animal procedures were performed according to the Italian national animal experimentation guidelines (D.L.116/92) upon approval of the experimental protocols by the Italian Ministry of Health’s Animal Experimentation Committee (authorization codes 0D183.0 and 0D183.1). Four-to-6-week-old female NOD.Cg-PrkdCSCsid Il2rgtm1Wjl/SzJ (NSG) mice (The Jackson Laboratory, Bar Harbor, ME, USA) were used.

### Assessment of Tumor Initiating Capability *In Vivo*

The main feature of stem cells is their ability, once implanted in a recipient mouse, to reproduce a tumor having the same phenotype of the original one. To evaluate this, 5 × 10^5^ cells were resuspended in 100 μl of a 1:1 growth medium/Matrigel (BD Biosciences) solution, and the cell suspension was injected subcutaneously into the flank of the animal. For each CSC line, five replicates of xenotransplants were done. For all the four lines, a tumor mass was detectable within 3–5 weeks in at least three of five mice. As soon as tumor mass reached a diameter of 10 mm, xenografts were explanted, and one-half of the mass was formalin-fixed, paraffin-embedded, and processed for histology to evaluate tumor phenotype in comparison with the parental human tumor. The other portion of tumors was dissociated into single cells that were seeded in tumor sphere medium and expanded to be assayed once again for stemness (CD44 and CD133 expression, ALDH activity).

### Histological Examination

Tumors were fixed with 10% formalin and paraffin embedded for histological analysis. Three-micrometer-thick sections were cut with microtome and automatically stained with hematoxylin and eosin (Ventana Symphony Stainer, JMD Histology and Histologistics Inc., Dudley, MA, USA).

Regression rate of tumor treated explanted xenografts has been evaluated according to the three-step regression system proposed by Junker et al. (46). Tumors were classified as grade I when no or only slight tumor regression was present; grade II were characterized by marked but incomplete tumor regression, grade II A with more than 10% vital tumor tissue, and grade II B with less than 10% vital tumor tissue. Grade III is assigned to cases with complete tumor regression without residual neoplastic cells.

### *In Vivo* Irradiation

Four- to 6-week-old female NSG mice (The Jackson Laboratory, Bar Harbor, ME, USA) were randomly assigned into four groups, one group for each CSC line. Each group was formed by 19 mice. For each line, CSCs were resuspended in 100 μl of 1:1 growth medium/Matrigel, and 5 ×10^5^ cells were injected subcutaneously in the flank of the animal. Tumor growth was measured twice weekly by an external digital caliper, and volumes were calculated using the following formula: π/6 × d2× D, where d and D represent shorter and longer tumor measurements, respectively. When tumors reached a dimension of 100–150 mm^3^, mice were randomized to control (three mice per group) and two treatment groups (eight mice per group). To validate *in vitro* results, mice were subjected to the LED and highest ineffective dose (HID) radiation identified with the assessment of *in vitro* sensitivity. LED was defined as the lowest dose that significantly impaired proliferation and increased apoptosis rate. HID was defined as the highest dose unable to exert a detrimental effect on CSCs proliferation and apoptosis *in vitro*. For CSCs that resulted resistant to all doses and for CSCs that resulted sensitive to all doses, the doses used *in vivo* were 5 and 10 Gy.

Because no differences have been observed in CSCs treated with different dose rates, the single-dose rate of 2,400 MU/min was used because it was the most safe and convenient due to its shorter time of administration.

On the day of radio-treatment, mice belonging to the treatment groups were placed into a plexiglass box where an anesthetic gas containing Vetflurane (Virbac, Barcelona, Spain) was insufflated. All the procedures, including image acquisition, contouring, elaboration of the treatments plan, and radio-treatment, were performed with the mouse inserted and immobilized inside the plexiglass cage. To this aim, mice were first subjected to computed tomography (CT) scan, and CT images were sent to the treatment planning system (TPS) dedicated for stereotactic radiotherapy treatments. The contouring of the volumes of interest was performed for target volume, spinal cord, heart, lungs, and bowel. At this point, treatment plans were elaborated, the plan consisting of two non-coplanar dynamic conformal arcs with optimized opening of MLC leaves based on dose constraints established during planning. Plan evaluation was performed carefully observing the dose distributions on each CT image and the dose–volume histograms, to check the radiation dose that reaches the target and the neighboring organs. For setup verification, we used the image-guided radiotherapy system “ExacTrac X-Ray 6D”, which allowed to carry out pre-positioning through the infrared system and positioning using the X-ray imaging system. Mice irradiation was performed by administering the maximum dose rate of 2,400 MU/min using the 10-MV FFF photon beam produced by the Varian Novalis-TrueBeamSTx linear accelerator. Thirty days after treatment, a CT scan was performed to verify tumor dimensions. Control animals were inoculated but not treated.

### Statistical Analysis

Quantitative endpoint (MTS, Annexin V and subcutaneous tumor masses volume) measured at different timepoints were evaluated, between groups of treatments and controls, using repeated measures ANOVA. Mean differences between start and end time point of sphere formation, proliferation, and apoptosis assays of all cell lines, highlighting *in vivo* behavior, are explored with a dimensionality reduction method [principal component analysis (PCA)] using “stats” (47) and “ggbiplot” (48) packages of R. All p-values have been Bonferroni corrected when multiple testing is used. Statistical analysis has been performed using R statistical environment.

## Results

### Lung CSCs Characterization

Three squamous cell carcinoma (SCC) and one adenocarcinoma (AC) lung CSC lines isolated from the corresponding lung cancer subtypes have been used in this study. All lung CSCs have been previously banked and fully characterized for stem cell properties and molecular alterations and used in our previous studies (38, 40). CD44 and CD133 molecules are two common surface markers used to isolate and identify CSCs. Flow cytometry analysis reveals high levels of CD44 for lines 229 and 136 (96.2% and 99%, respectively), whereas the levels of CD133 are very low (4% and 2.2%, respectively). On the contrary, line 36 presents high CD133 level (90%) and very low CD44 expression (0.8%). In line 196, CD133 was not detectable, whereas CD44 was present at 52%.

The SRY-box transcription factor 2 (SOX2) is considered a core regulator of stemness (49), and its expression was also evaluated in lung CSCs by immunoblot. In particular, line 36 expresses high levels of Sox2 protein, whereas lines 196 and 229 express only basal level of these proteins. Line 136 showed no detectable level of Sox2 protein.

Increased ALDH activity has been described in putative stem cells from different carcinomas (32, 50–53). ALDH activity was tested by flow cytometry using the ALDEFLUOR assay. Such assay revealed a negative activity in lines 229 and 196, a high activity in line 36, whereas a medium activity was found in line 136 (**Table 2**).

Molecular markers described above were assessed to provide a molecular overview of the starting cellular material. Even if these markers have been associated with stem cells properties, the definition of CSC status do not rely on their expression but on the demonstration of cells self-renewal and potency (38, 40).

The mutational status of epidermal growth factor receptor (EGFR), P53, and Kirsten rat sarcoma virus (KRAS) was also analyzed; EGFR and P53 resulted wild type (WT) in all the four cell lines, whereas KRAS was mutated in lines 229 and 196 (**Table 2**).

### Lung CSCs From Different Patients Show Different *In Vitro* Sensitivity to Radiotherapy Protocols

To evaluate radiation-induced cytotoxic activity on the putative cells responsible for lung cancer growth and spreading, we established lung CSC cultures, which represent the optimal cellular targets for radiation therapy. In the present study, we investigated the cytotoxic effect of different radiation doses (5, 8, 10, and 20 Gy), administered at different dose rates (600, 1,400, and 2,400 MU/min) to lung tumor sphere cultures.

**Figure 1:**
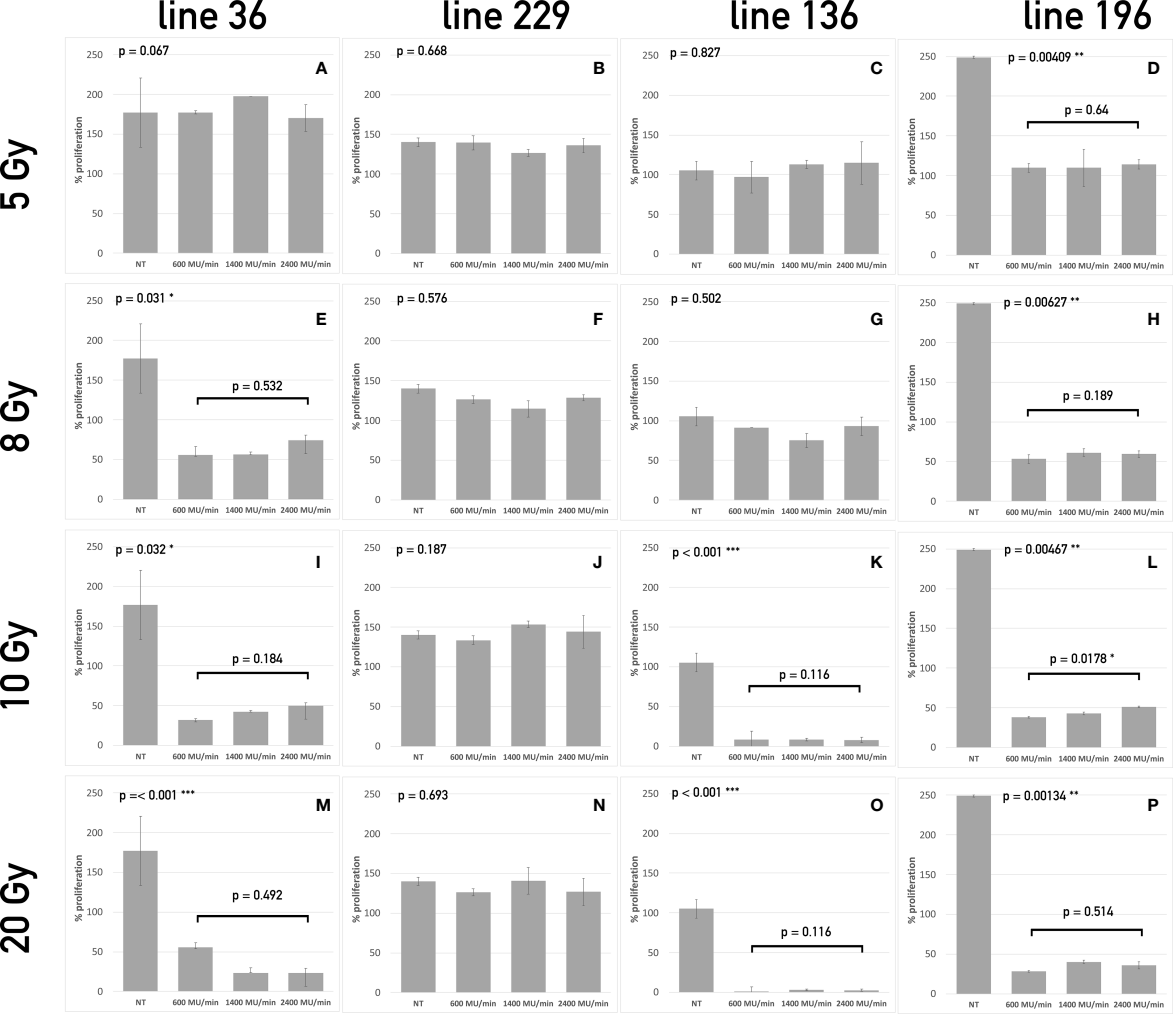
Proliferation assay of the four CSCs line treated with different dose/dose rate combinations. The bars represent cell proliferation of lines 36 **(A, E, I, M)**, 229 **(B, F, J, N)**, 136 **(C, G, K, O)**, and 196 **(D, H, L, P)** treated with 5 Gy **(A–D)**, 8 Gy **(E–H)**, 10 Gy **(I–L)**, and 20 Gy **(M–P)** compared to untreated cells at 14 days after treatment. For each dose, three different dose rates have been used. On the top left corner of each graph, the p-value of the repeated measure ANOVA test used to assess differences between treated and untreated cells. When significant differences exist, the p-value of the repeated measure ANOVA test between treatments (dose rates) is also reported. (* symbols are used to summarize the number of 0 digits after the decimal sign in significant results. * p < 0.05, ** p < 0.001, *** p < 0.001).

**Figure 2:**
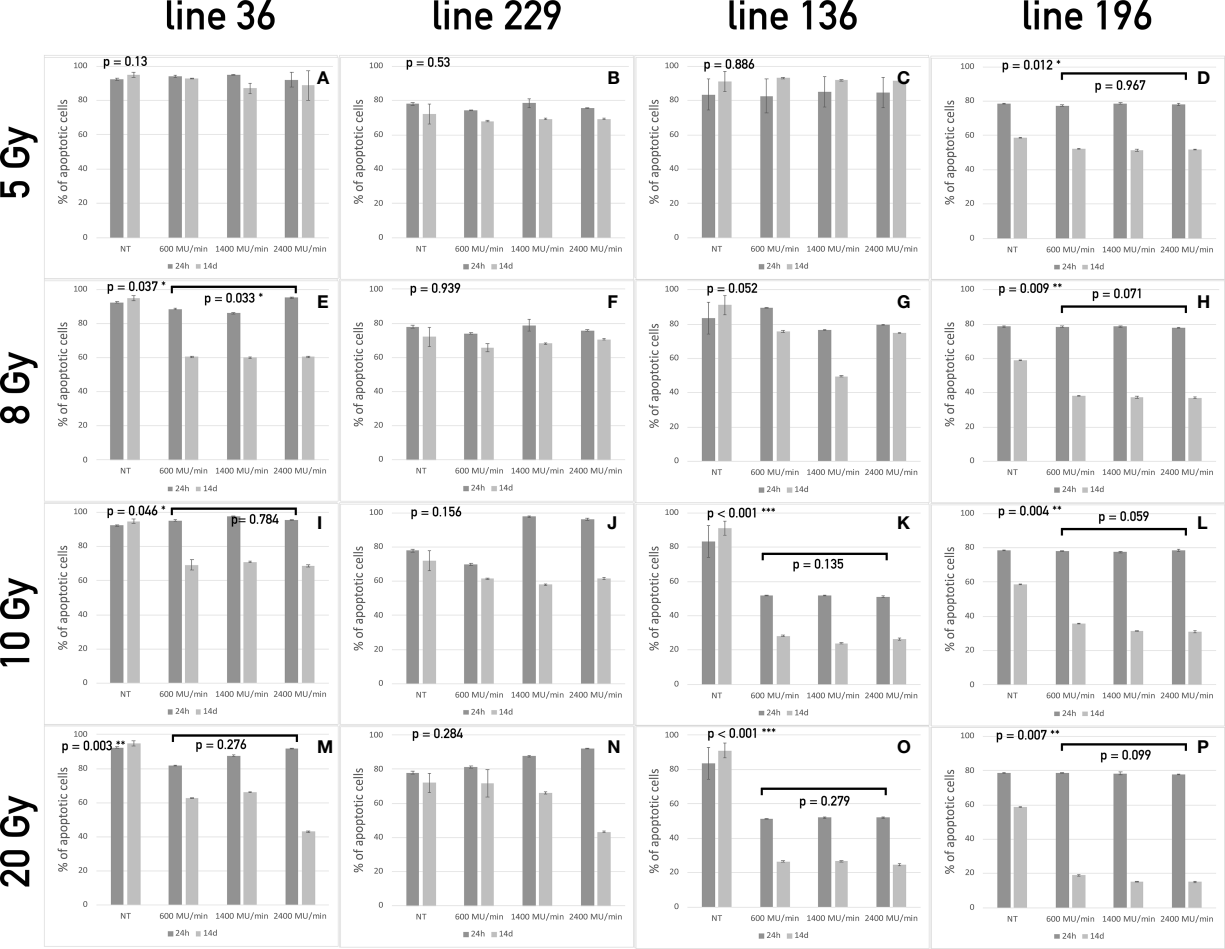
Apoptosis assay of the four CSCs line treated with different dose/dose rate combinations. The bars represent the percentage of apoptotic cells in lines 36 **(A, E, I, M)**, 229 **(B, F, J, N)**, 136 **(C, G, K, O)**, and 196 **(D, H, L, P)** treated with 5 Gy **(A–D)**, 8 Gy **(E–H)**, 10 Gy **(I–L)**, and 20 Gy **(M–P)** compared to untreated cells at 24 h and 7 days after treatment. For each dose, three different dose rates have been used. On the top left corner of each graph, the p-value of the repeated measure ANOVA test used to assess differences between treated and untreated cells. When significant differences exist, the p-value of the repeated measure ANOVA test between treatments (dose rates) is also reported. (* symbols are used to summarize the number of 0 digits after the decimal sign in significant results. * p < 0.05, ** p < 0.001, *** p < 0.001).

To evaluate the effect of the radiotherapy treatment *in vitro*, different assays, including proliferation, apoptosis, and sphere formation assays, were performed at different times (24 h, 48h, 72 h, 7 days, and 14 days) after treatment administration. On day 14, the four different cell lines displayed different behavior at the diverse doses tested. On the contrary, no differences were found among the different radiation dose rates in terms cell proliferation or apoptosis (**Figure 2**), except for line 196 treated with a dose of 10 Gy. In particular, line 229 resulted resistant at all doses tested; indeed, no significant differences emerged in the proliferation potential compared to the control group (not treated, NT) (**Figures 1B, F, J, N**). On the contrary, a completely opposite behavior was seen in line 196 (**Figures 1D, H, L, P**), which resulted highly sensitive to all different treatments in comparison with the NT group. A significant reduction in the proliferative potential was already evident at the lowest dose of 5 Gy, with an increasing trend with dose increase.

The LED for line 36 was 8 Gy because no significant effect could be observed below this value (**Figures 1A, E, I, M**). At 10 and 20 Gy, the percentage of proliferating cells was further reduced. Line 136 showed a significant decrease in proliferation starting from 10 Gy (**Figure 2C, G, K, O**), whereas, at the lower doses, no significant differences were evident among treated and not treated groups.

Twenty-four hours after radiation treatment, all CSC lines showed only a modest increase in apoptosis, whereas, on day 14, a heterogeneous apoptotic behavior is observed in different conditions in a way similar to what observed for proliferation. In all condition assessed, dose rate did not influence the apoptotic response. Line 36 resulted sensitive to doses starting from 8 Gy with a decrease in the percentage of viable cells ranging from 40% to 50% after 14 days (**Figures 2A, E, I, M**). The difference among dose rates at the total dose of 8 Gy was significant (**Figure 2E**), but the overall percentage of viable cells was similar in the three groups of treatment after 14 days. This suggests that the difference may not be biologically relevant for long-term response. No difference was found between treated and control cultures of CSCs line 229 (**Figures 2B, F, J, N**). This confirms the complete refractoriness to radiation of this line, as already indicated by the proliferation assay. Line 136 responded to treatment starting from the dose of 10 Gy (**Figures 2K, O**), with a noticeable increase in apoptotic cell population. Line 196 confirmed to be sensitive to all doses with increasing induction of apoptosis with the increase of dose (**Figures 2D, H, L, P**). Again, no difference was observed between different dose rates.

Both proliferation and apoptosis assays revealed for each CSCs line the existence of a specific sensitivity threshold. It was possible to identify for each line the LED, defined as the smallest amount of radiation required to exert a statistically significant effect on both proliferation and apoptosis. Consequently, HID was defined, for each line, as the highest dose of radiation unable to trigger any significant effect on proliferation or apoptosis. Thus, the following values were calculated as follows: Line 1: 5 Gy (HID) and 8 Gy (LED); line 2: 20 Gy (HID) and LED not found; line 3: 8 Gy (HID) and 10 Gy (LED); line 4: 5Gy (LED) and HID not found.

### Sphere Formation Assay

**Figure 3:**
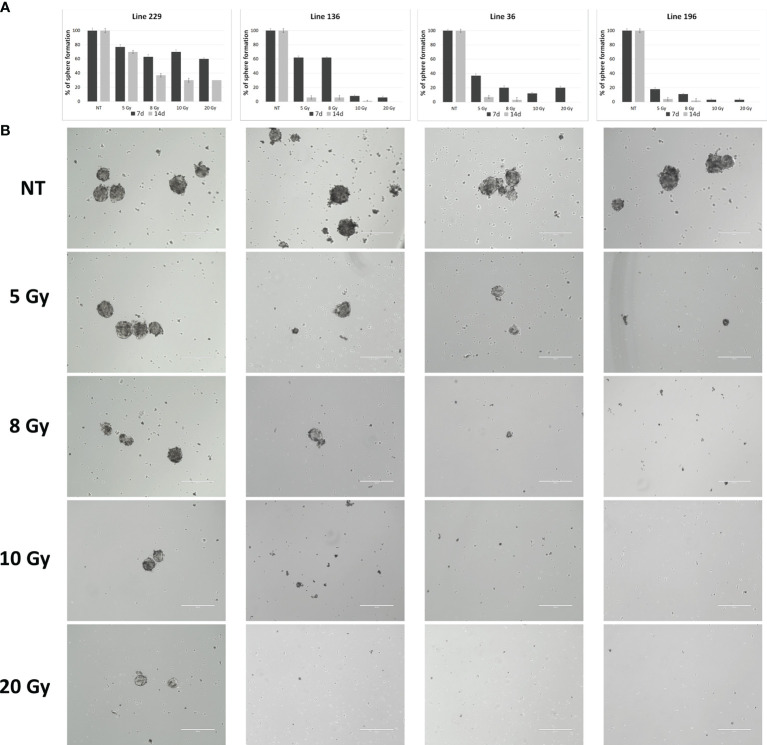
Sphere formation assay. **(A)** Quantitative representation of sphere formation capabilities of the four lines after treatment with different doses (number of spheres per FOV after treatment/number of spheres per FOV before treatment, four replicates for each dose/line). **(B)** Representative images of sphere formation ability.

Line 229 exhibited a slight reduction in the ability to give rise to new cell clusters after 7 days from radiation, settling around 20% at 5 Gy up to 40% at 20 Gy; after 14 days, this reduction was a little more marked reaching a reduction around 70% at highest doses (10 and 20 Gy). On the contrary, line 196 after 7 days showed a strong reduction already at 5 Gy (80%) that reached almost 100% at 10 and 20 Gy; after 7 more days, the ability to give rise to new spheres was almost abolished already at 5 Gy. Line 36 had a behavior similar to the line 196, showing about 70% reduction in sphere-forming ability after 7 days at 5 Gy and around 80% at the superior doses; after 14 days, this reduction was almost 100% for all doses. Line 136 revealed a 40% reduction after 7 days at 5 and 8 Gy, reaching 100% at 10 and 20 Gy; after 14 days, the reduction was almost 100% at all doses (**Figures 3A, B**).

In conclusion, the *in vitro* experiments demonstrated that CSCs derived from individual patients show different sensitivity to the radiation treatment, although no relevant differences were observed among the different dose-rate protocols (600, 1,400, and 2,400 MU/min) nor between 10 and 20 Gy doses in all the cell lines analyzed.

### CSCs Isolated From Lung Cancer Tissues Are Able to Initiate Tumor Growth in Mice

**Figure 4:**
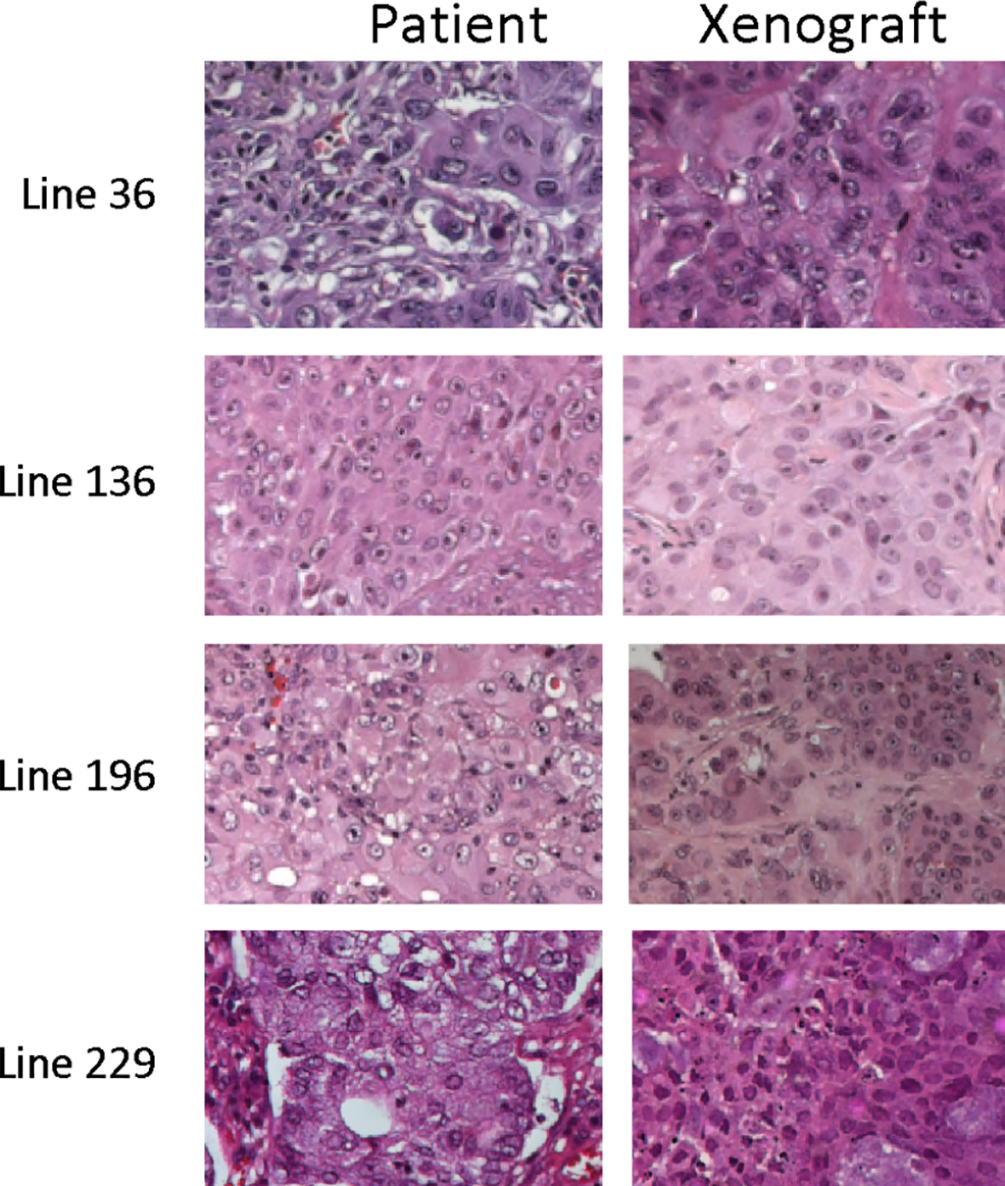
Representative images of the histology of tumor xenograft and of corresponding patient tumors. Hematoxylin and eosin images of matching tumor tissue from parental patient tumors and explanted CSC-derived tumor grafts.

*In vitro* experiments have already demonstrated that CSCs derived from different lung cancer patients present different sensitivity to radiotherapeutic treatments. To investigate *in vivo* sensitivity, CSCs derived from patients’ lung carcinoma were expanded *in vitro* and injected in the flank of female Nod Scid Gamma (NSG) mice. All injected animals generated subcutaneous masses 1 to 3 months after injection. The histology of the explanted tumors was similar to that of the corresponding original patients’ tumors (**Figure 4**)

When tumors reached a diameter of about 100–150 mm^3^, animals were subjected to CT scan to better estimate tumor dimensions and to exactly define the zone to expose to radiotherapy.

CSCs from different lung samples showed an *in vivo* sensitivity to radiotherapy protocols comparable to that found in the *in vitro* treatments.

Animal models were subjected to two different radiotherapeutic protocols, which had shown different efficacy in *in vitro* experiments. In particular, the *in vivo* treatment involved the use of the lowest among the effective doses and the highest dose among those that did not give a response. Moreover, because all cell lines showed no significant difference in the *in vitro* response between 10 and 20 Gy, the maximum dose used *in vivo* was 10 Gy, to reduce animal toxicity.

The radiation doses administered for each experimental group were the following: 5 and 8 Gy for line 229, 5 and 10 Gy for line 196, 5 and 10 Gy for line 36, and 8 and 10 Gy for line 136.

**Figure 5:**
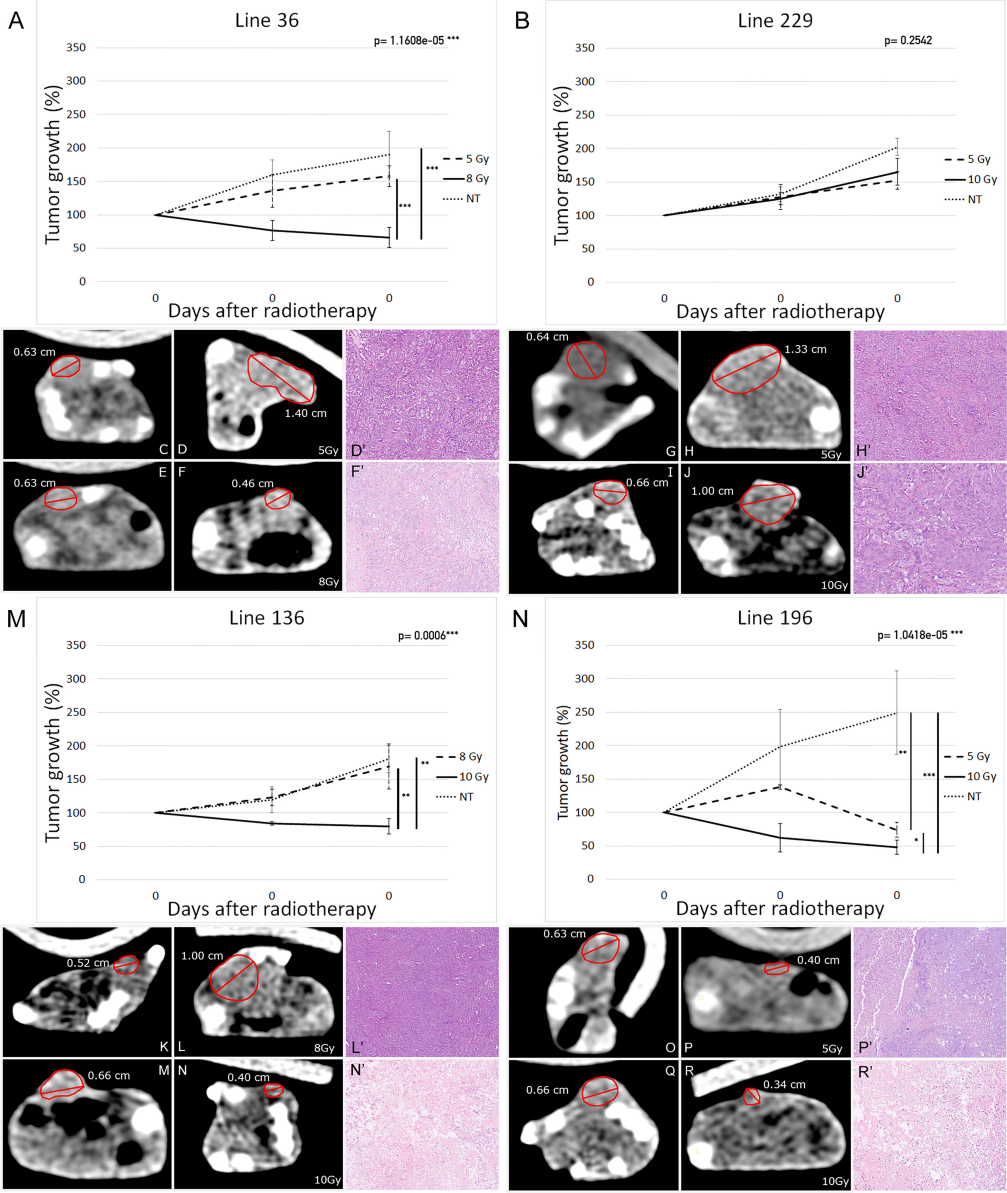
Tumor xenografts’ volume variation in response to radiotherapy (19 mice per group: eight mice for each of the treatment group and three mice for the NT group). Percentage-volume variation 15 and 30 days after treatment in lines 36 **(A)**, 229 **(B)**, 136 **(M)**, and 196 **(N)**. Highest Ineffective Doses represented as dashed lines, lowest effective doses represented as continuous lines, and untreated controls represented as dotted lines. Representative CT images before **(C, E, G, I, K, M, O, Q)** and 30 days after treatment **(D, F, H, J, L, M, P, R)** in lines 36 **(C–F)**, 229 **(G–J)**, 136 **(K–M)**, and 196 **(O–R)** at highest ineffective dose **(D, H, L, P)** and lowest effective dose **(F, J, N, R)**. Repeated measures ANOVA p-values are reported on top right of each graph. Vertical bars indicate statistically significant differences between groups of treatment. (*p < 0.05, **p < 0.01, ***p < 0.001).

Tumor growth was evaluated by external caliper measurement at the onset of radiotherapy and after 15 and 30 days. At the end of this period, mice were subjected to a final CT scan to exactly evaluate the tumor mass reduction in comparison with the initial CT scan. The *in vivo* results corresponded to those obtained *in vitro*. In particular, the radiation treatment was quite ineffective for line 229 tumor grafts as shown in **Figure 5**, where the trend of tumor growth for the two treated groups was comparable to the NT group.

On the contrary, line 196 tumor grafts displayed a high sensitivity to radiotherapy, revealing a steady decrease of the initial tumor mass. Indeed, not only treated mice did not present tumor growth after treatment but also the tumor mass was reduced up to 50% with 10 Gy dose. Mice treated with 5 Gy showed only an arrest in tumor growth without evident mass reduction. In NT mice, the tumor mass reached a volume about two times larger than the initial one (**Figures 5N, R–O**).

In agreement with the *in vitro* data, the other two cell lines showed an intermediate behavior. Indeed, mice injected with line 36 and treated with 10 Gy showed a significant reduction of about 20% of the initial tumor mass, whereas 5-Gy treatment did not induce a tumor mass reduction in comparison with the control group (**Figures 5A**, **C**–**F**). In the same way, line 136 mice treated with 10 Gy showed an arrest in tumor growth together with a decrease in tumor mass of about 30%; whereas the 8-Gy treatment was unable to induce tumor growth arrest in comparison with the control group (**Figures 5M, K–N**).

Histological evaluation of the pulmonary carcinomas of the explanted xenografts confirms the response rate observed using CT. Various morphologic patterns of tumor regression have been observed: targeted foci of central eosinophilic coagulative necrosis often surrounded by atypical cells (**Supplementary Figure 1A**), vascular granulation tissue and fibrosis (**Supplementary Figure 1B**), and loose connective tissue (**Supplementary Figure 1C**). Occasionally, calcifications were reported. No foams cell infiltration and multinuclear giant cells were observed as described in human NSCLC samples after neoadjuvant therapy (54). In all untreated tumors, we did not appreciate any sign of regression and therefore were classified as grade I. The same morphological pattern was observed for line 229 treated with 5 or 10 Gy and for lines 36 and 136 treated with HID (**Figures 5D, H, J, L**). Partial tumor regression with more than 10% of residual neoplastic component, coherent with tumor regression grade IIA (**Figure 5F**) has been observed in about one-third of the tumors corresponding 196 cells treated with 5 or 10 Gy and lines 36 and 136 treated with LED. The remaining one-third of these treated tumors showed a significant regression, with areas of necrosis representing around 50%–60% of the tumor bed (**Figures 5N', R'**).

**Figure 6:**
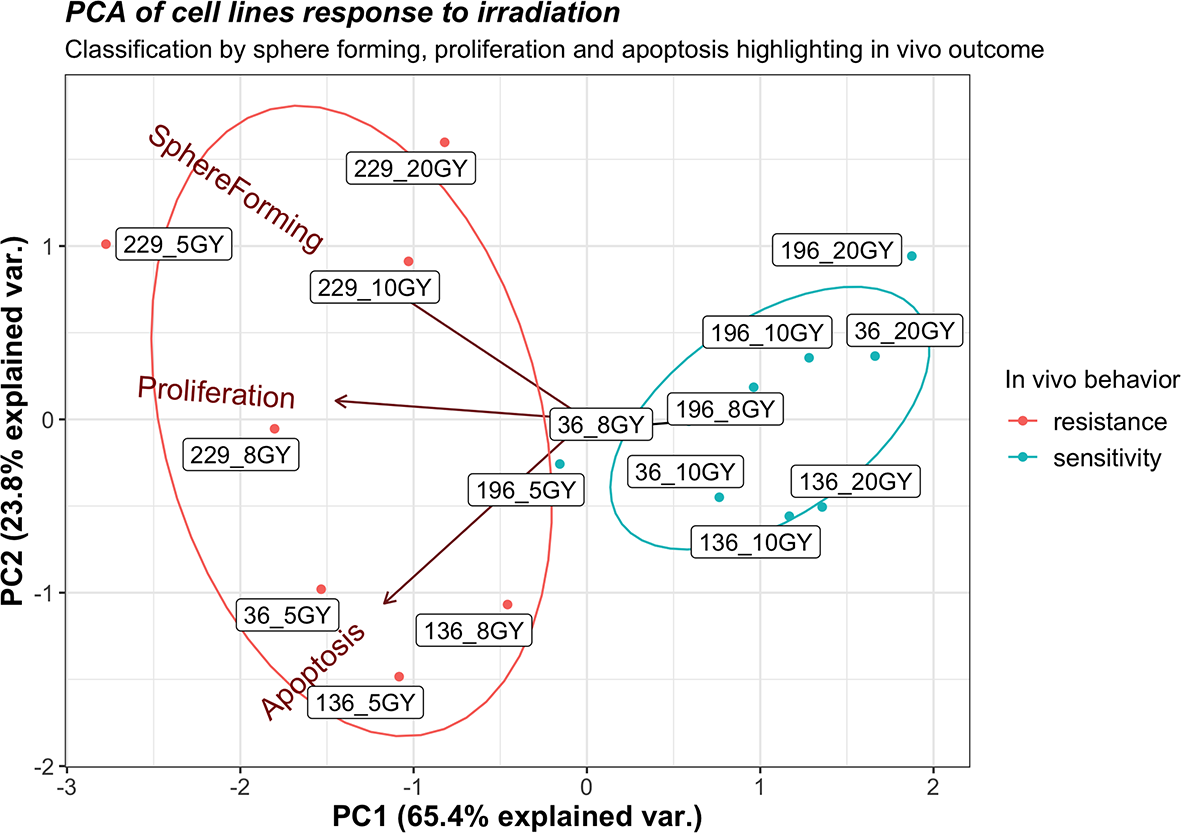
Principal component analysis for sphere formation, proliferation, and apoptosis of all cell lines exposed to 2,400 MU/min at different gray (Gy) with *in vivo* response annotation. The label of each point identifies the cell line and the administered radiation dose (e.g., 196_10 identify line 196 exposed to 10 Gy).

Furthermore, a PCA of sphere formation, proliferation, and apoptosis assays (**Figure 6**) strengthens the experimental outcomes. The acute angles between the axes of sphere formation and proliferation or proliferation and apoptosis are signs of the correlations between these variables; on the contrary, sphere formation and apoptosis are less correlated. Tumor lines used for *in vivo* assay, which demonstrates sensitivity to radiotherapy, clusters together (196_5, 196_10, 36_8, and 136_10), as well as those lines that show resistance (229_5, 229_10, 36_5, and 136_8). The analysis also shows that values of sphere formation, proliferation, and apoptosis are useful for classifying even those cell lines not tested *in vivo* that show the same behavior as the one tested. A borderline case is 196_5 Gy that is close to the resistance cluster but shows a small response *in vivo*. Then, cell lines showing sensitivity *in vitro* group together as resistant ones, highlighting how the clusters replicate *in vivo* behavior.

## Discussion

Patient-derived CSCs have been characterized both *in vitro* and *in vivo* to evaluate the existence of an intrinsic resistance to radiotherapy. Data have shown that the level of resistance to radiation greatly varies among CSCs ranging from a complete resistance to all doses, exhibited by line 229, to the resistance to lower doses, exhibited by lines 36 and 136, to a radiosensitive cellular phenotype exhibited by line 196, which responds even at the lower employed dose. In particular, the most sensitive CSCs were affected by the treatment with a dose as low as 8 Gy, whereas other cultures showed a response only at higher doses (10 and 20 Gy). Only in one case, the treated CSCs showed a complete resistance to all tested treatments suggesting that, in some cases, radiotherapy may not be sufficient as monotherapy. Reproducibility of results among replicates indicates that the LED can be identified for each CSC culture. It is interesting to note that line 196, which is the most responsive to irradiation, shows a weak expression of stem cell markers, except for CD44. Although the identification of molecular characteristics that are predictive of sensitivity to radiation is out of the scope of this manuscript, this evidence may suggest that stem cells markers may be associated with therapeutic response. To evaluate stem cells markers association with therapeutic outcome, specific investigation will be set up in the future.

Furthermore, it has been shown that the dose rate does not affect the cellular response to treatment. The *in vivo* treatment of tumors obtained from subcutaneous xenograft of NSG mice showed that the sensitivity profile corresponded to the one observed *in vitro*. In fact, each of the animals was treated using two different doses based on the *in vitro* results. The lowest dose that induced cell death and proliferation arrest *in vitro* and the highest dose among those that did not produce a response *in vitro* were used. These two doses, identified for each of the lines used, were defined as minimum effective dose and maximum ineffective dose. In all treated animals, there was a significant reduction in tumor mass when the animals were treated with the LED. Conversely, when the animals were treated with the MID, the corresponding tumors did not undergo a volume reduction. These results demonstrate that the *in vitro* model is predictive of the treatment in mouse xenografts. This evidence supports the application of an *in vitro* only predictive platform for the support of clinical decision. Although patient-derived animal models require different months to be established, CSC cultures may be obtained and assessed for radiosensitivity within 1 month. This makes the assay suitable for clinical use because the time required is compatible with the related standard of care. In particular, the identification of the LED is extremely important for the reduction of radiotoxic side effects that greatly limit the benefit of curative radiotherapy. Although these results are supportive of this hypothesis, a clinical study is required to clearly define the translational setting. On one side, the use of a single-dose *in vitro* irradiation may be sufficient to estimate the amount of the total effective dose; on the other side, dose fractionation should be specifically planned to treat corresponding patients.

It is also important to note that the presented model is missing an appropriate evaluation of the effects that, in treated patients, is exerted by the immune system. The fundamental role of immune system in therapeutic response of radio treated lung cancer patients stimulated the interest of scientific community. It has been demonstrated that radiation-induced immune effects may be both immunostimulatory and immunosuppressive. The lack of large-scale studies on those phenomena leads to the absence of a widely accepted common consensus about the role of the immune system in lung cancer patients treated with radiotherapy. It is clear, anyway, that the underlying mechanisms must be elucidated to achieve a comprehensive view of their therapeutic implications.

It has been reported that SABR may have toxic consequences ranging from mild effects like fatigue and transient esophagitis to severe ones like pneumonitis, chest wall pain, and rib fractures (55, 56). These toxic effects are dependent on both the dose used and the volume of the treated area. Because SABR has optimal local control rates and a favorable toxicity in comparison with surgical procedures, its use is increasing. This is particularly important in early stage, node negative NSCLC when surgical therapies are risky or unfeasible. These observations sustain the recent focus of lung SABR strategies toward the limitations of toxicity, even in the treatment of complex cases, thus expanding the range of applications for lung cancer. For this reason, it is very important to identify patient-specific factors associated with SABR eligibility to tailor treatment protocols and support patient management. Moreover, for a complete and sustained long-term response, it is critical to identify treatments that are able to target CSCs that are responsible for progression and relapse. Our results suggest that the sensitivity profile of CSCs is individually linked to a specific cellular phenotype that confers coherent properties to their differentiated tumor progeny. The lack of a decrease in volume, observed in subcutaneous tumors generated from radioresistant CSCs, is coherent with the role of CSCs in the sustainment of the cancerous dynamics. These progenitor cells constitute a proliferative reservoir for differentiated tumor cells that inherit treatment resistance. The identification of a specific threshold dose that is effective in the induction of CSC apoptosis is, therefore, mandatory for a long-term control of NSCLC.

In this work, the individual sensitivity to *in vitro* radiotherapy of CSCs, isolated from lung cancer patients, was evaluated. Although it is well established that a hallmark characteristic of CSCs is their resistance to radiation therapy, the delivery of high doses of radiation overcomes this resistance. The identification of the LED needed to kill CSC is therefore useful to balance efficacy and dose-dependent toxic effects. Different combinations of doses and dose rates were used. It has been demonstrated that the minimal amount of effective radiation varies from patient to patient. It has been also demonstrated that the dose rate does not affect the response, suggesting that the increase in frequency used to administer the treatment does not produce a relevant benefit. Nevertheless, treatments administered to higher dose rates are safer because their administration requires a much shorter exposure. Our data suggest that the efficacy of higher dose rates is comparable with the one of conventional treatment, thus supporting their use for faster and safer treatment.

The main evidence, supported by the presented data, is that the level of resistance to radiation therapy of cancer cells is intrinsic and not influenced by environmental conditions. The evidence that cells respond to the same LED in both *in vitro* cultures and *in vivo* tumors, which are completely different environment, indicates an intrinsic, patient-specific phenomenon. This suggests that sphere-forming cell cultures are representative of tumor sensitivity by themselves. The clinical translation of these observation requires more issues to be clarified: the influence of chemotherapy in combined treatment, the impact of dose fractionation in several sessions of treatment, or the biological substrate of this differential response of CSC are some of them. Another important issue for clinical translation is the availability of tumor tissues as starting material for CSC isolation, which is not feasible for non-surgical patients with the actual procedures. Nevertheless, the reported knowledge is a fundamental step for a deeper comprehension of the radiobiology of CSC in a real-life model. The investigation of such models in a clinical setting, moreover, may confirm the hypothetical clinical benefit of a patient-tailored treatment. The administration of the LED, specifically identified for each tumor, at the higher dose rate may thus contribute to lower the toxic effects of the treatment while maintaining the highest level of efficacy for an optimal treatment.

## Data Availability Statement

The raw data supporting the conclusions of this article will be made available by the authors, without undue reservation.

## Ethics Statement

The studies involving human participants were reviewed and approved by Institutional Review Board of ARNAS Civico of Palermo. The patients/participants provided their written informed consent to participate in this study. The animal study was reviewed and approved by Ministero della Salute, Italy.

## Author Contributions

Conceptualization: AG and SF. Data curation: CP and SF. Formal analysis: SF and EM. Funding acquisition: AG. Investigation: CP, RG, GB, SI, FC, PM, GF, and CC. Methodology: CP, GB, GS, and AE. Writing—original draft: RG. Writing—review and editing: AL and SF. All authors contributed to the article and approved the submitted version.

## Funding

This research was funded by the Italian Ministry for Industrial Development (MISE), project title: “Sviluppo di trattamenti innovativi di radioterapia personalizzata, basati sulla sensibilità indi-viduale delle cellule staminali tumorali”, grant number F/0157/00/X26 - CUP B68C15000180008, Decreto del Ministero dello sviluppo economico n. 5922 of 11.11.2015.

## Conflict of Interest

The authors declare that the research was conducted in the absence of any commercial or financial relationships that could be construed as a potential conflict of interest.

## Publisher’s Note

All claims expressed in this article are solely those of the authors and do not necessarily represent those of their affiliated organizations, or those of the publisher, the editors and the reviewers. Any product that may be evaluated in this article, or claim that may be made by its manufacturer, is not guaranteed or endorsed by the publisher.
