# Supplementary material for: Ex Vivo Irradiation of Lung Cancer Stem Cells Identifies the Lowest Therapeutic Dose Needed for Tumor Growth Arrest and Mass Reduction In Vivo
Source: Front Oncol. 2022 May 12;12:837400. doi: 10.3389/fonc.2022.837400 (PMC9133629; doi:10.3389/fonc.2022.837400)
Supplement: Supplementary file 1 [file DataSheet_1.docx]

Supplementary Material


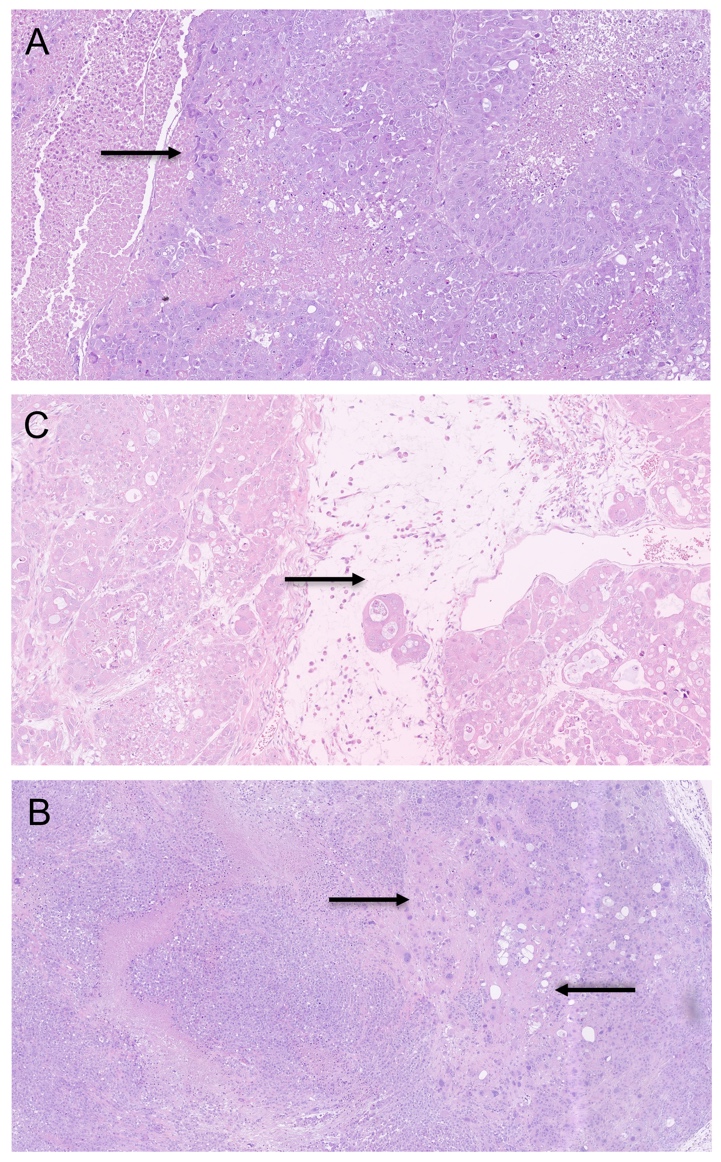


**Supplementary Figure 1.** Observed morphologic patterns of tumor regression. **A.** Targeted foci of central eosinophilic coagulative necrosis often surrounded by atypical cells. **B.** Vascular granulation tissue and fibrosis. **C.** and loose connective tissue.
